# Engaging residents to choose wisely: Resident Doctors of Canada resource stewardship recommendations

**Published:** 2019-03-13

**Authors:** Justin Hall, Reza Mirza, James Quinlan, Evan Chong, Karen Born, Brian Wong, Christopher Hillis

**Affiliations:** 1 Division of Emergency Medicine, Department of Medicine, University of Toronto, Ontario, Canada; 2 Division of Internal Medicine, Department of Medicine, McMaster University, Ontario, Canada; 3 Division of Internal Medicine, Department of Medicine, Memorial University of Newfoundland, Newfoundland, Canada; 4 Department of Family and Community Medicine, University of Toronto, Ontario, Canada; 5 Institute of Health Policy, Management and Evaluation, University of Toronto, Ontario, Canada; 6 Division of Internal Medicine, Department of Medicine, University of Toronto, Ontario, Canada; 7 Department of Oncology, McMaster University, Ontario, Canada

## Abstract

**Background:**

Resident doctors are integral to healthcare delivery in Canada. Engaging residents in resource stewardship is important for professional development, but also as they are drivers of healthcare resource use. To date, no national resident-specific resource stewardship guideline has been developed. Resident Doctors of Canada (RDoC) in collaboration with Choosing Wisely Canada (CWC) sought to develop an evidence-informed, consensus-based list of five recommendations to promote resource stewardship.

**Methods:**

RDoC convened a taskforce with diverse geographic and specialty representation to develop candidate recommendations targeting resident resource stewardship behaviours using a consensus-based process, supported by a literature review. Residents across the country provided feedback on the candidate recommendations via an online questionnaire. The taskforce used this feedback to finalize the list.

**Results:**

The taskforce prepared 28 candidate recommendations for consideration. A detailed literature review and consensus process narrowed this list to 12 candidate recommendations for consultation. A total of 754 residents (754/10,068 residents = 7.5%) representing all provinces and levels of residency training reviewed and ranked the candidate recommendations. The highest-ranked recommendations comprised the final list.

**Conclusion:**

Resident doctors are willing and able to demonstrate leadership in advancing resource stewardship by the development of a national resident-specific list of Choosing Wisely Canada recommendations.

## Introduction

There is international recognition of the need to reduce unnecessary medical tests, treatments, and procedures,^1^^-^^5^ and the explicit expectation that physicians develop resource stewardship proficiency during training.^6^^-^^8^ Recent estimates suggest up to 30% of all healthcare in Canada is potentially unnecessary and can cause harm to patients.^5^ In 2015, the Royal College of Physicians and Surgeons of Canada released revised CanMEDS competencies (CanMEDS 2015 Physician Competency Framework), to be used by all physicians in Canada, which added resource stewardship requirements as part of the leader competencies.^6^ Yet, postgraduate medical education programs in both the United States and Canada have been slow to integrate resource stewardship education into training. Residents are front-line clinicians and provide direct patient care with graduated levels of autonomy. They are often the first and last physician whom patients encounter in academic hospitals through intake and discharge.^9^ Residents fulfill an important role in the healthcare workforce and cover patient care around the clock in hospital and primary care settings. Emerging data suggest that trainees have higher rates of resource utilization than practicing physicians.^10^^,^^11^ Therefore, education to advance residents’ resource stewardship knowledge has the potential to both address current healthcare resource utilization and shape future physician practices.

Choosing Wisely Canada (CWC), launched in 2014, is a clinician-led campaign that promotes resource stewardship by developing evidence-based recommendations to reduce unnecessary tests and treatments.^12^ Recently, CWC partnered with the two Canadian medical student associations – the Canadian Federation of Medical Students and the Fédération médicale étudiante du Québec - to publish a list for medical education.^13^ While this list of recommendations holds relevance for residents in their role as learners, it does not speak to the role that residents play as care providers. Further, the CWC medical education list development process did not involve residents, a missed opportunity to raise awareness of resource stewardship among residents.

Resident Doctors of Canada (RDoC) is a not-for-profit organization that represents over 10,000 resident doctors across Canada. Residents connected to the four Québec medical schools are represented by the *Fédération des médecins résidents du Québec*. RDoC engages in advocacy on behalf of residents and collaborates with other national health organizations to foster excellence in training, wellness, and patient care. RDoC collaborated with CWC to lead a consensus process to generate a list of recommendations that identifies tests, treatments, or procedures that are unnecessary, potentially harmful to patients, and relevant to all Canadian residents’ education and future practice. This article describes the development of the CWC list of recommendations for residents.

## Methods

The RDoC Practice Committee is a 16-member resident committee that supports the delivery of patient-centred care and transitions from residency training to independent practice through Committee recommendations and advocacy efforts. The Social Accountability Working Group is a subgroup of seven residents from the RDoC Practice Committee; five of these residents, with geographic and specialty diversity (four specialties, three provinces, two with advanced training in quality improvement, patient safety, and health policy), comprise the taskforce responsible for the CWC list development. Specifically, this taskforce was responsible for leading the consensus and evidence review process. The McMaster University Research Ethics Board deemed this work exempt from full review.

### Consensus process for RDoC CWC list generation

**Figure 1:**
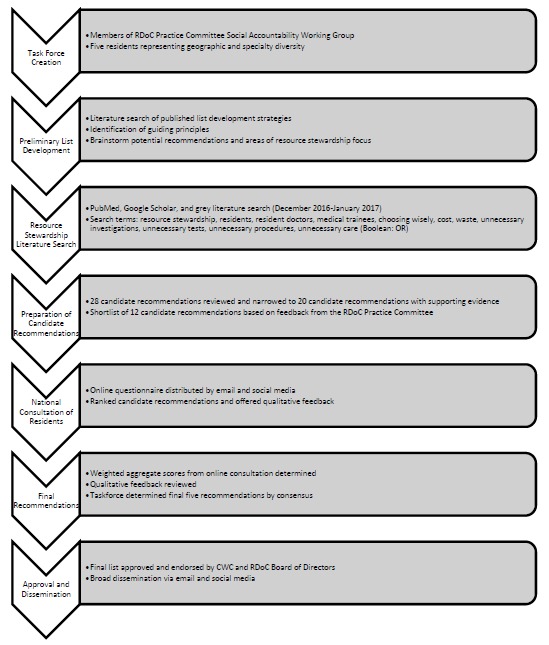
Overview of the development process for the Resident Doctors of Canada (RDoC) Choosing Wisely Canada (CWC) List “Five Things Residents and Patients Should Question”

The taskforce established its iterative consensus-based list development process (see Figure 1) on the CWC medical education list approach,^13^ as well as five published strategies from specialty-specific projects.^14^^-^^18^ To begin, the taskforce established by consensus a set of six overarching principles to guide the development of candidate (proposed) recommendations grounded in the published literature and CWC’s operational principles for list development.^14^^-^^18^ Each candidate recommendation must: 1) arise frequently in residency training, 2) have relevance to residents, 3) play a role in shaping future behaviours, 4) be one that residents may feasibly address during their training, 5) focus on residents’ use of tests, treatments or procedures, and 6) contribute to building a more economically sustainable, cost-conscious healthcare system.

To ensure relevance for residents, from both generalist and specialist training programs, candidate recommendations had to address broad issues related to residency education and practice, rather than specific clinical conditions. The taskforce generated the initial list of candidate recommendations by searching PubMed, Google Scholar, and the grey literature for English-language resource stewardship articles relevant to resident education and practice (see Figure 1 for search terms) and meeting to discuss further candidate recommendations. The taskforce presented the initial list of candidate recommendations to the RDoC Practice Committee at a meeting and by email. The RDoC Practice Committee provided additional suggestions that were not captured by the initial review and discussion process. The taskforce updated the candidate recommendation list, including supporting evidence, to reflect the RDoC Practice Committee suggestions.

The RDoC Practice Committee then reviewed the revised list of candidate recommendations to remove redundant items, ensure alignment with the six overarching principles of list development, and assess the evidence base to support inclusion (see Appendix A). The taskforce put forward a shortlist of 12 candidate recommendations for national consultation (see Table 1) with Canadian resident physicians through an online questionnaire (Gravity Forms, Rocketgenius Incorporated, Virginia Beach, Virginia). The questionnaire (see Appendix B) provided background information to describe the overall purpose of developing the CWC list. Residents ranked the candidate recommendations and could provide comments and suggestions for additional recommendations. The English-language questionnaire was distributed in April 2017 through the provincial residency organizations’ email listservs and social media (Facebook and Twitter). Respondents had the opportunity to enter a draw to win an Apple Watch as an incentive to complete the survey.

**Table 1:** Candidate Recommendations for the Choosing Wisely Canada List for Residents, as Rated by 754 Residents Representing all Provincial Residency Organizations, April-May 2017. The total weighted score and aggregate rank are provided, and the five final recommendations are bolded.

Candidate Recommendation	Total Score	Aggregate Rank
**Don’t order investigations that will not change your patient’s management plan**.	7050	1
**Don’t order repeat laboratory investigations on inpatients who are clinically stable**.	6312	2
Don’t allow transitions of patient care without proper colleague handover, including active issues, pertinent or pending investigations, and contingency plans.	5969	3
**Don’t order intravenous (IV) when an oral (PO) option is appropriate and tolerated**.	5791	4
**Don’t order non-urgent investigations or procedures that will delay discharge of hospital inpatients**.	5729	5
**Don’t order invasive studies if less invasive options are available and as effective**.	5003	6
Don’t involve a specialty/consulting service without having a specific clinical question in mind.	4795	7
Don’t order continuous monitoring unless necessary.	4455	8
Don’t sign prewritten order sets without carefully evaluating if each investigation is indicated for the patient.	4222	9
Don’t prescribe or order brand name medications if an equivalent, less expensive generic alternative is available.	3263	10
Don’t ask junior learners to order investigations without ensuring their understanding of the need for each specific test.	3147	11
Don’t contribute to a culture of cost ignorance in residency education.	2998	12

To generate a rank-ordered list of recommendations, we calculated a weighted score and aggregate rank for each recommendation using the results of the online consultation by awarding 12 points for each number 1 resident rank and downwards to 1 point for each number 12 resident rank, with the points summed to create the weighted score. The taskforce reviewed the rank-ordered list and narrative comments to inform the final list of five recommendations. After the online consultation with residents and for each of the five final recommendations, we conducted a detailed literature review to capture any new studies that were published since the initial literature search. PubMed, Google Scholar, and the grey literature were searched in May 2017 with each combination of one search term from a) and b) [a) resource stewardship, residents, resident doctors, medical trainees, choosing wisely, cost, waste (Boolean: OR) AND b) unnecessary investigations, unnecessary tests, laboratory tests, laboratory investigations, unnecessary procedures, unnecessary care, management plan, intravenous medication, oral medication, hospital discharge, delayed discharge, invasive studies, clinical stability, clinically stable (Boolean: OR)]. CWC faculty (CH, KB, BW) reviewed the final list of recommendations to ensure that the wording and format of the recommendations were consistent with the CWC campaign. The RDoC Board approved and endorsed the list of resident recommendations in June 2017. It was publicly released on the CWC website in July 2017 (see https://choosingwiselycanada.org/residents/).

## Results

The taskforce generated 16 candidate recommendations which were augmented by 12 additional suggestions from the RDoC Practice Committee. Of these 28 candidate recommendations (see Appendix A), eight were removed as they were redundant with other recommendations or lacked evidence. For example, we removed “Don’t ask junior learners to seek patient consent for procedures that are unfamiliar to them” due to a lack of supporting evidence specific linking this recommendation to resource stewardship and removed “Don’t admit a patient to hospital before code status has been documented” because it overlapped with “Don’t forget to discuss goals of care early on in a hospital admission.”

The RDoC Practice Committee reviewed the revised list of 20 candidate recommendations and supporting evidence from the literature search (see Appendix A) to ensure alignment with CWC’s sixprinciples of list development. Eight additional candidate recommendations were removed at this stage. For example, “Don’t forget to follow-up on results in a timely manner” was removed as it did not align with principles 5 and 6. The resulting list of 12 candidate recommendations went forward for national consultation (see Table 1).

A total of 754 of the 10,068 Canadian residents completed the consultation(response rate = 7.5%). Residents from all provinces with English-language medical schools provided input (Newfoundland (n=61, 8.1%), Nova Scotia (n=56, 7.4%), Quebec (n=27, 3.6%), Ontario (n=407, 54.0%), Manitoba (n=60, 8.0%), Saskatchewan (n=49, 6.5%), Alberta (n=43, 5.7%), and British Columbia (n=51, 6.8%)). Respondents were in their first (n=215, 29%), second (n=209, 28%), third (n=148, 20%), fourth (n=97, 13%), fifth (n=56, 7%), or other (n=29, 4%) year of residency training. In addition to the ranking of the candidate recommendations, respondents offered narrative comments; 102 general comments and 70 suggestions for additional recommendations were received (Table C1 in Appendix C). The narrative comments broadly related to four key themes: a) wording considerations for the suggested recommendations; b) process considerations (e.g., imbalance between inpatient and outpatient recommendations); c) operationalizing consideration/suggestions for implementation of recommendations; and d) general comments of support for the suggested recommendations. Within the additional recommendations suggested by the respondents, there were none that aligned with all six principles and were supported by evidence. No new recommendations were added as a result of the consultation.

Table 1 summarizes the results of the consultation, by weighted score and aggregate rank. While ranked as the third highest recommendation, CWC suggested to remove: “Don’t allow transitions of patient care without proper colleague handover, including active issues, pertinent or pending investigations, and contingency plans.” This recommendation did not conform with the format of the CWC recommendations, which are supposed to be worded as negative statements (e.g., things that clinicians should question) and not phrase positive recommendations in negative language. The final list of recommendations was comprised of the highest-ranked candidate recommendations, with the exception of the one addressing transitions. Table 2 summarizes the results of the literature review supporting the list of recommendations.

**Table 2:** Final Top 5 Recommendations for the Choosing Wisely Canada List for Residents with supporting evidence

Recommendation	Evidence
*Don’t order investigations that will not change your management plan*.	Investigations should be ordered to establish a diagnosis, monitor therapy, or screen for diseases for which patients are at a sufficient risk. Investigations are often ordered that will not impact management. Residents order more screening investigations than attending physicians with a study of Canadian family medicine residents showing that residents ordered an average of 3.3 to 5.7 more inappropriate screening tests per sample patient.^19^ For example, an asymptomatic woman under 40 with no family history who is concerned about breast cancer should not receive a screening mammogram as the incidence of breast cancer is low and screening does not offer a mortality benefit.^20^ In situations where there is a low risk of serious illness, screening or diagnostic tests offer little reassurance to patients, or resolve symptoms.^21^ On the other hand, high-risk patients may warrant treatment irrespective of the test result. To illustrate, thrombophilia testing in patients with an unprovoked pulmonary embolism at high risk for recurrence is not helpful since these patients should receive indefinite anticoagulation, regardless. In these cases, laboratory thrombophilia evaluation generally provides little information that improves a management decision and the testing itself carries potential patient risks.^22,23^
*Don’t order repeat laboratory investigations on inpatients who are clinically stable*.	Observational studies suggest that resident doctors order routine daily CBC (complete blood count) and electrolyte panels more frequently than attending physicians.^24-26^ Importantly, daily phlebotomy contributes to patient discomfort and iatrogenic anemia.^27-29^ Hospital-acquired anemia secondary to phlebotomy is linked to worse health outcomes, mortality, and need for transfusion in the setting of acute myocardial infarction,^29-31^ trauma,^32^ and intensive care patients.^33-35^ Studies support the safe reduction of repetitive laboratory investigations without a negative impact on patient outcomes, including readmission rates, critical care utilization, adverse events, or mortality.^27,28,36,37^
*Don’t order intravenous (IV) when an oral (PO) option is appropriate and tolerated*.	Patients are often ordered IV medications when PO options are available, appropriate, and equally bioavailable. A common example is antibiotics that are highly orally bioavailable^38-40^ (e.g., fluoroquinolones, trimethoprim-sulfamethoxazole, clindamycin, metronidazole, and fluconazole) with randomized controlled trials demonstrating non-inferiority between oral and intravenous antibiotics for uncomplicated diverticulitis,^41,42^ severe and non-severe pneumonia in adults,^43-46^ and acute pyelonephritis.^47^ A recent study of resident doctors in the Netherlands showed that 94% of residents were unaware of international guidelines to switch from IV to PO antibiotics in clinically stable hospitalized patients with community-acquired pneumonia on day three of treatment.^48^ Aside from the increased cost of IV medications, they increase potential for harm as peripheral catheters increase the risk of complications, including extravasation, infections, decreased mobility, and thrombophlebitis.^49,50^
*Don’t order non-urgent investigations or procedures that will delay discharge of hospital inpatients*.	Discharges of hospital inpatients are commonly delayed for investigations that will not change acute management. Examples include performing thoracoscopic lung biopsies,^51-53^ imaging to investigate incidental findings,^54-57^ non-urgent assessment by a specialist,^58,59^ waiting for bloodwork or imaging results,^60-62^ or echocardiography for patients with mild heart failure.^63^ Delayed discharges contribute to hospital crowding and negatively impact care efficiency.^64,65^ Crucially, longer lengths of stay are a risk factor for nosocomial infections, venous thromboembolism, pressure injuries, immobility, adverse drug reactions, malnutrition, and deconditioning.^65-69^ Outpatient investigations should be arranged when possible, if good follow-up can be assured.
*Don’t order invasive studies if less invasive options are available and as effective*.	When deciding on investigations or treatments for patients, it is prudent to consider the least invasive option that will have similar sensitivity and specificity to guide clinical decision making while minimizing potential harm. To illustrate, when diagnosing acute appendicitis in children, ultrasound should be considered before CT scanning. Not only is ultrasound radiation- and contrast-free, but it is equivalent to CT scanning in the diagnosis and management of acute appendicitis.^70-73^ Another example is conducting a urea breath test rather than endoscopy to prove *H. pylori* eradication. The sensitivity and specificity of the urea breath test are superior compared to other diagnostic tests and the risk of patient harm is minimal.^74,75^

## Discussion

RDoC partnered with CWC to develop a list of recommendations specific to resident doctors to help residents identify and reduce unnecessary tests, treatments and procedures in clinical training environments. RDoC first led a rigorous consensus-building process to engage residents from across Canada to inform list development and then conducted a detailed evidence review to support the recommendations. While Choosing Wisely campaigns are active in more than 20 countries worldwide, RDoC, in partnership with CWC, is the first national organization representing resident doctors to develop and publish a Choosing Wisely list.^76^

Building on the CWC list for medical education,^13^ the list for residents helps to advance trainee-led conversations about the need for resource stewardship content in postgraduate medical education. Unlike medical students, residents in academic teaching hospitals can order investigations and treatments independently. Therefore, a resident-specific list that involved residents in the consultation process and that reflected their multiple roles as teachers, trainees, clinicians, and drivers of healthcare resource use, is important. Resident engagement through a resident-led consultation process was critical to RDoC’s list development process. We reached over 750 residents through this effort, and this list may demonstrate an innovative means to engage residents in resource stewardship advocacy and to potentially identify and reduce unnecessary tests, treatments and procedures in their own training environments and future practices.

To further promote uptake, CWC and RDoC are distributing the list broadly in English and French. This includes the CWC app, CWC and RDoC websites, social media channels such as Facebook and Twitter, residency program directors and chief residents, and partner organizations such as the Royal College of Physicians and Surgeons of Canada and the College of Family Physicians of Canada. We hope for integration at the level of the residency training programs given the CanMEDS 2015 Physician Competency Framework requirements for resource stewardship as part of the CanMEDS leader competencies.^6^ There is growing engagement of learners and trainees in Choosing Wisely campaigns internationally,^77^^,^^78^ and this list can serve as a starting point for other national resident associations to engage in campaigns. The intention of this list is to serve as a conversation starter for residents and trainees as well as staff physicians about resource stewardship. The list may also be used by other health professions programs to develop complementary frameworks that may be integrated into other clinical learning environments such as nursing, and pharmacy, and allow for thoughtful, patient-centred resource stewardship discussions across health professions.

While list development is an important first step, it must be implemented into the clinical environment to change practice.^79^^-^^82^ Even more importantly, the residency training environment needs to be transformed and residents can and want to be part of the solution. First, as part of teaching rounds, residents may serve as role models for junior trainees by bringing forward cases of overuse and how harm may ensue from unnecessary testing.^83^ Second, as residents’ future ordering behaviours and resource use are strongly influenced by their training environment,^10^^,^^11^ residents can lead resource stewardship initiatives to change test ordering practices to reduce utilization.^84^ Finally, resident-led projects may serve as a catalyst for larger system-level change by promoting evidence-informed practices and shifting ordering and management practices of those around them.^85^ Indeed, recent research suggests that implementation of Choosing Wisely campaign recommendations can lead to reductions of unnecessary tests.^86^^,87^ A formal evaluation strategy examining resident list use, incorporation into residency training programs, and effect on patients has not been finalized at the time of publication.

There are multiple limitations of the current work. The low resident response rate (7.5%) to the online questionnaire may limit the representativeness of the aggregate rankings. Despite the response rate, the survey is supported by a relatively large number of narrative comments, particularly in comparison with some of the recommendations from specialty societies. A second limitation is that the survey was only available in English, and it is possible that French-speaking resident participation may have been limited. The final list was translated into French and disseminated widely in both languages. A third limitation is the lack of demographic data about the survey respondents. While we collected school and year of training for each participant, we do not have information on training program, speciality, or primary practice setting to preserve anonymity of respondents from smaller programs and schools. While the RDoC Practice Committee and taskforce reflects specialty and geographic diversity, we are unable to comment on the proportion of generalist and specialist respondents or the proportion of hospital, office, or community-based training environments. Last as outlined above, list development is an important first step, but implementation into the clinical environment is necessary to change practice. The current work was not designed to assess or evaluate behaviour or practice changes, and therefore, an important next step would be to examine if and how these recommendations influence ordering practices in different clinical environments.

### Conclusion

The CWC list of recommendations for residents demonstrates leadership of Canadian residents for advancing resource stewardship training and competencies. Resident doctors play an important role in healthcare in Canada as trainees, providers of patient care, and drivers of resource use in their clinical decision making. By joining the CWC campaign with this list of recommendations, Canadian resident doctors are helping to advance conversations on resource stewardship in medical education as well as drive improvements to healthcare systems by reducing overuse.

## Appendix A

**Resident Doctors of Canada (RDoC) Candidate Recommendations as identified by the RDoC Practice Committee.**

**Redundant recommendations have been shaded in grey.**

1. Don’t order investigations that will not change your patient’s management plan.
2. Don’t order repeat laboratory investigations on inpatients who are clinically stable.
3. Don’t allow transitions of patient care without proper colleague handover, including active issues, pertinent or pending investigations, and contingency plans.
4. Don’t order intravenous (IV) when an oral (PO) option is appropriate and tolerated.
5. Don’t order non-urgent investigations or procedures that will delay discharge of hospital inpatients.
6. Don’t order invasive studies if less invasive options are available and as effective.
7. Don’t involve a specialty/consulting service without having a specific clinical question in mind.
8. Don’t order continuous monitoring unless necessary.
9. Don’t sign prewritten order sets without carefully evaluating if each investigation is indicated for the patient.
10. Don’t prescribe or order brand name medications if an equivalent, less expensive generic alternative is available.
11. Don’t ask junior learners to order investigations without ensuring their understanding of the need for each specific test.
12. Don’t contribute to a culture of cost ignorance in residency education.
13. Don't add additional routine or screening labs to blood draws, if not indicated.
14. Don’t forget to follow-up on results in a timely manner.
15. Don't keep patients on unnecessary isolation precautions.
16. Don’t order tests for educational purposes alone.
17. Don’t miss an opportunity to learn about specialty-specific resource stewardship.
18. Don’t initiate an in-hospital subspecialty referral if a patient is followed by the subspecialty elsewhere or the referral can be arranged in the community.
19. Don’t forget to discuss goals of care early on in a hospital admission.
20. Don’t use hospital equipment (i.e. printers, dressings, scrubs, etc.) for personal use.
21. Don’t ask junior learners to seek patient consent for procedures that are unfamiliar to them.
22. Don’t admit a patient to hospital before code status has been documented.
23. Don’t wear hospital-issued attire outside of the hospital.
24. Don’t order tests simply because your preceptor may want them.
25. Don’t refer a patient to an outpatient specialist/consultant if they are already followed by the same type of specialist.
26. Don’t repeat lab work without reviewing previous results.
27. Don’t order new imaging studies if patients have received them at another site.
28. Don’t miss an opportunity to discuss test costs with junior learners.

## Appendix B

**Resident Doctors of Canada’s Choosing Wisely Canada Resident List Details for National Online Consultation**

Background:

Up to 30% of tests and treatments that physicians order, are unnecessary, do not change patient care management, and may cause patient harm. Resource stewardship is an approach which focuses on appropriate allocation of resources for patient care which considers benefits, harms, and overall costs.

Resident Doctors of Canada has partnered with Choosing Wisely Canada to create a preliminary list of 12 recommendations for residents. We hope that this list will help guide residents on the appropriate use of healthcare resources in residency.

**WE WANT YOUR INPUT** to create a final list of “Five Things Residents Should Question”.

By filling out this short 5-minute anonymous consultation, you will be a part of this movement to reduce unnecessary care. To participate, please click on the following link: Resource Stewardship Consultation. This survey will close on May 12.

If you complete this survey you will be automatically entered into a draw to win an Apple Watch. Please provide your email address if you wish to be considered in this draw.

Should you have any additional questions regarding this project, please do not hesitate to contact us. Thank you for your help in this important initiative!

Contact Us:

**Website:** EN: www.choosingwiselycanada.org FR: http://www.choisiravecsoin.org

**Email:** info@choosingwiselycanada.org

**Phone Number:** 1-416-864-6060 x 77548

**Facebook:** https://www.facebook.com/ChoosingWiselyCanada

**Twitter:** @ChooseWiselyCA @ChoisirAvecSoin

Please click the box to begin.

By clicking “I Agree”, you confirm that the information obtained from the consultation will be used for purposes of list development by Resident Doctors of Canada and Choosing Wisely Canada. This consultation is completely anonymous and voluntary.

Demographics:

1. Which school are you currently attending?
  Dalhousie University
  McGill University
  McMaster University
  Memorial University of Newfoundland
  Northern Ontario School of Medicine
  Queen’sUniversity
  Université Laval
  Université de Montréal
  Université de Sherbrooke
  University of Alberta
  University of British Columbia
  University of Calgary
  University of Manitoba
  University of Ottawa
  University of Saskatchewan
  University of Toronto
  Western University
  I prefer not to disclose
2. What year of residency are you in?
  1
  2
  3
  4
  5
  6
  7
  Other (please specify):
  I prefer not to disclose

Criteria for List Evaluation:

The goal of this resident list is to highlight important issues that arise during residency training that may affect how residents behave with respect to resource stewardship (consideration of the benefits, harms, and overall costs). The recommendations aim to highlight the unique role of resident doctors as health care providers, trainees, and teachers across disciplines. Please evaluate the recommendation statements based on the following criteria:

1. **Frequency** – commonly occurs in residency training
2. **Relevance** – problem that residents may relate to
3. **Impact** – influences future behaviors of residents and has potential to improve quality of care
4. **Feasibility** – issue that residents have the power to address during their training
5. **Appropriateness** – relevant to patient management and contributes to building a more cost-conscious healthcare system

Choosing Wisely Canada Resident Consultation List:

Please identify your top 5 recommendations:

1. Don’t order investigations that will not change your patient’s management plan.
2. Don’t order repeat laboratory investigations on inpatients who are clinically stable.
3. Don’t allow transitions of patient care without proper colleague handover, including active issues, pertinent or pending investigations, and contingency plans.
4. Don’t order intravenous (IV) when an oral (PO) option is appropriate and tolerated.
5. Don’t order non-urgent investigations or procedures that will delay discharge of hospital inpatients.
6. Don’t order invasive studies if less invasive options are available and as effective.
7. Don’t involve a specialty/consulting service without having a specific clinical question in mind.
8. Don’t order continuous monitoring unless necessary.
9. Don’t sign prewritten order sets without carefully evaluating if each investigation is indicated for the patient.
10. Don’t prescribe or order brand name medications if an equivalent, less expensive generic alternative is available.
11. Don’t ask junior learners to order investigations without ensuring their understanding of the need for each specific test.
12. Don’t contribute to a culture of cost ignorance in residency education.

Comments (optional):

Please suggest any additional recommendations (optional):

## Appendix C

**Table C1:** Narrative Comments received from the national online consultation of residents during the development process of the Resident Doctors of Canada’s Choosing Wisely Canada List organized by theme with illustrative quotations as examples

Theme	Illustrative Quotations
***Support for Current Approach*** -Applicable to a range of generalist and specialist disciplines -All candidate recommendations important -Adopt evidence-based medicine approach	-“Very difficult to rank these as many are equally important/valuable.” -“The above-mentioned recommendations are excellent. I could relate to each of them- well done!” -“All of these are equally as important.” -“If I could put 10 stars beside ‘Don’t order investigations that will not change your management plan’ I would. Tests should not be ordered unless we are going to do something with the information. Tests beget more tests… and more costs.” -“Important to understand and appreciate that the nature of our health care system is there are limited resources, given our model of socialized medicine, that unfortunately we always have to find the best bang for our buck.” -“This is a great initiative! Very useful recommendations.”
***Operationalizing Recommendations*** -Include in postgraduate medical education teaching -Incorporate into electronic medical record ordering systems -Make costs of tests and medications readily available to providers and patients -Educate patients, staff physicians, and other health professionals about the recommendations	-“Teaching related to the actual costs of each lab test, diagnostic test, and procedure should be regularly taught in medical school, residency, and CME.” -“I do not see any reason why the ACTUAL COST of each test, investigation, medication, consult, ‘day spent in hospital bed’, is not readily available to doctors and residents if they wish to see it. In fact, I do not see why the ACTUAL COST of the aforementioned services and medications are not readily available to patients (if they are interested)! Trying to navigate through healthcare economics is needlessly difficult, and this makes striving towards responsible resource allocation nearly impossible.” -“There really is NOT enough discussion about healthcare costs in Residency Education. This has to change.” -“Electronic systems can potentially help facilitate many of these changes.” -“Physicians should be required to write in or select a ‘justification’ while ordering various tests (mandatory field could be added to EMR orders). If they have not thought of a valid reason to be ordering the test, they shouldn’t be placing the order. Emergency/critical care patients may be an exception.” -“Residents have a general lack of knowledge when it comes to cost of patient care tests. In particular, medical imaging is widely over ordered like blood work, without consideration of its high cost. Residents should see it be educated in the expenses of investigations.”
***Process Considerations*** -Include specific recommendations related to treatments rather than broad considerations -Incorporate more outpatient examples -Exercise caution around limiting investigations in training environments	-“If you are going to make recommendations they should be specific, as are many of the specialty societies’ recommendations are, as opposed to broad sweeping generalized statements.” -“As far as letting junior learners order investigations; part of the learning process in medicine is thinking through a process and ordering tests to help confirm or refute what you think. Ordering tests is an important part of this but often many diagnoses can be made without a complete workup. Part of residency/an academic environment is to do the complete workup and understand why you are ordering each test. As you become more senior and eventually staff you learn which tests you need to order for certain things and which ones can be omitted. I think if we start to become to restrictive as far as ‘necessary tests’ a major component of the learning process maybe lost.” -“I would appreciate auto-substitutions from Pharmacy for less expensive or generic medication alternatives.” -“As a Family medicine resident, this list seems a bit too impatient-centered, and seems to me as if it ignores the other 50% of residents who spend most of their time in outpatient clinics (I know that was likely not your intention, it just seems that way!). I think the issues can be fixed by just thinking about what about outpatient measures residents can take, such as ensuring that all patients are screened according to guidelines, doing their best to solve polypharmacy in seniors by discontinuing medications, or ensure that goals of care and advanced care planning have been addressed.”
***Wording Considerations*** -Consider “do” statements rather than “don’t” statements	-“It would be useful if this list had at least a couple of ‘do’ statements instead of only ‘do not’; tone is vital to get resident buy-in.” -“Perhaps phrasing everything as ‘don’t’ is not an ideal way to foster compliance and ownership of these ideas.” -“Change wording to the positive (or don’t start everything with “don’t”, as it sounds punitive. E.g., Only order continuous monitoring if necessary, etc.”
***Other Recommendations*** -Suggestions for additional recommendations	-“Don’t supplement with IV fluids if patient stable and can swallow safely.” -“Don’t order urinalysis in asymptomatic patients.” -“Always round on patients early and perform evaluation for discharge planning to avoid unnecessary delays in discharging patients home (planning for home care and appropriate discharge location).” -“Don’t forget to have a code discussion with every patient upon admission to hospital.” -“Don’t routinely order daily investigations that will not change your management.”
